# Stimulus Size Modulates Periodic and Aperiodic EEG Components in SSVEP-Based BCIs

**DOI:** 10.3390/brainsci16040424

**Published:** 2026-04-18

**Authors:** Gerardo Luis Padilla, Fernando Daniel Farfán

**Affiliations:** 1 Neuroscience and Applied Technologies Laboratory (LINTEC), Bioengineering Department, Faculty of Exact Sciences and Technology (FACET), National University of Tucuman, Superior Institute of Biological Research (INSIBIO), National Scientific and Technical Research Council (CONICET), Av. Independencia 1800, San Miguel de Tucumán 4000, Argentina; 2 Institute of Bioengineering, Universidad Miguel Hernández of Elche, 03202 Elche, Spain

**Keywords:** BCI, SSVEP, EEG, aperiodic activity

## Abstract

**Highlights:**

**What are the main findings?**

• Increasing stimulus size selectively enhances the attended SSVEP response without affecting the unattended stimulus in a competitive BCI paradigm.
• Stimulus size modulates the aperiodic (1/f) spectral slope under specific attentional and frequency conditions.

**What are the implications of the main findings?**

• The results reveal a dual mechanism in which stimulus size influences both oscillatory and background neural activity.
• These findings provide insights for optimizing stimulus design in non-invasive SSVEP-based brain–computer interfaces.

**Abstract:**

**Background/Objectives:** Steady-State Visual Evoked Potential-based Brain–Computer Interfaces face a critical trade-off between system accuracy and user visual fatigue. To address this challenge, the objective of this study was to determine how the spatial manipulation of stimulus size modulates the full spectral dynamics of the Electroencephalogram, encompassing both the periodic oscillatory response and the aperiodic (1/f) background noise. **Methods:** Twenty-two healthy subjects completed a sustained visual attention task using a competitive stimulus paradigm (20 Hz and 30 Hz) presented in three spatial dimensions (Small, Medium, and Big). Parieto-occipital brain signals were decomposed using the spectral parameterization algorithm (SpecParam) to extract frequency-specific visually evoked response power and the aperiodic slope, while visual fixation was continuously monitored via eyetracking. **Results:** Increasing stimulus size induced a statistically significant gain in the power of the attended signal (Target) without increasing the response of the peripheral distractor. Simultaneously, larger stimuli produced a significant increase in the aperiodic slope during 20 Hz attention and visual rest, suggesting increased cortical inhibition and a reduction in broadband neural activity. This aperiodic modulation was not observed at 30 Hz. **Conclusions:** The improvement in Signal-to-Noise Ratio with increasing stimulus size arises from a dual neurophysiological mechanism: enhancement of the periodic evoked response together with a reduction in background neural noise.

## 1. Introduction

Steady-State Visual Evoked Potential (SSVEP)-based Brain–Computer Interfaces (BCIs) have established themselves as one of the most promising modalities for non-invasive communication and external device control. Due to their high Information Transfer Rate (ITR) and minimal user training requirements, SSVEP-based BCIs are among the most widely used paradigms for non-invasive communication and device control [1,2]. However, the practical implementation of these interfaces outside the laboratory faces a persistent challenge: the critical trade-off between system accuracy and user comfort. While it is well established that certain stimulation parameters, such as frequencies in the low-to-medium range and high luminance contrasts, elicit the most robust neural responses, these same factors contribute significantly to visual fatigue and discomfort during prolonged use [3]. This fatigue is not merely a subjective nuisance; it induces quantifiable neurophysiological changes, such as increased alpha/theta activity and a decreased Signal-to-Noise Ratio (SNR), which degrade system performance over time [4]. Consequently, optimizing the visual interface design has become a priority to maximize the neural response without compromising visual ergonomics.

Among the various properties of the visual stimulus that modulate the SSVEP response, stimulus size—or the visual angle it subtends on the retina—has proven to be a fundamental determinant of signal power. Classical and contemporary literature agree that larger stimuli recruit more extensive neuronal populations in the primary and extrastriate visual cortex, resulting in significantly larger SSVEP amplitudes [1,5]. Seminal studies have established that reducing stimulus size leads to a drastic decrease in SNR and fundamental frequency power, suggesting the existence of a minimum size required for reliable classification [6,7]. Furthermore, recent research has corroborated that larger stimuli not only elicit stronger Electroencephalographic (EEG) responses but also provoke a more pronounced pupillary constriction, indicating a higher level of sensory processing [5]. However, despite the evidence favoring the use of large stimuli to maximize the periodic oscillatory signal (the SSVEP peaks), a gap persists in understanding how stimulus size affects the “background” of the EEG signal. Historically, it has been assumed that increasing the stimulation area might introduce irrelevant noise or peripheral distractions, but few studies have quantified whether the spectral environment in which the SSVEP resides is positively or negatively altered by the dimensions of the stimulus.

To address this issue, it is necessary to adopt a new analytical paradigm that transcends the exclusive evaluation of periodic oscillations. Over the last decade, the aperiodic component of the EEG power spectrum—the background activity that follows a 1/f distribution—has ceased to be considered mere physiological noise, and the spectrum is now recognized as a critical functional marker of brain state [8]. Theoretical models and empirical evidence suggest that the spectral slope (or aperiodic exponent) reflects the Excitation/Inhibition (E/I) balance in the underlying neural circuits. Specifically, a steeper slope (a faster power decay at higher frequencies) has been interpreted as reflecting changes in the excitation–inhibition balance and a reduction in asynchronous neuronal firing rates, effectively “cleaning” the neural background noise [9]. Modern methodological tools, such as the spectral parameterization algorithm (SpecParam, formerly Fitting Oscillations and One Over F, FOOOF), now allow for the mathematical separation of these aperiodic components from rhythmic oscillations, avoiding the confounding of broadband power changes with true oscillatory activity [10].

The integration of aperiodic component physiology into BCI design raises a novel hypothesis regarding the impact of stimulus size. A recent study has demonstrated that the intensity of sensory stimulation not only modulates the evoked response but also alters aperiodic dynamics. Specifically, it has been observed that more intense visual stimulation induces a steeper spectral slope in the visual cortex [11], a finding interpreted as a homeostatic mechanism of post-stimulus functional inhibition to counteract incoming excitation. Extrapolating this finding to the context of SSVEP-BCIs, we assume that the larger stimuli use could induce a dual benefit: increasing the power of the periodic signal (SSVEP) through retinotopic recruitment, and simultaneously evoking greater cortical inhibition reflected by a steeper aperiodic slope. If confirmed, this would suggest that large stimuli do not necessarily introduce peripheral noise, but promote a more stable background neural activity instead, potentially improving the overall SNR through inhibitory mechanisms.

Therefore, the present study investigates how stimulus size modulates both periodic and aperiodic components of the EEG spectrum during an SSVEP-based BCI task. To address this question, EEG signals were decomposed into oscillatory and aperiodic contributions using spectral parameterization. This approach allows us to examine the spectral environment in which SSVEP responses emerge, moving beyond traditional analyses focused exclusively on frequency peaks. Overall, our findings suggest that the influence of stimulus size extends beyond a simple amplification of the periodic response, pointing instead to a broader modulation of EEG spectral dynamics. Understanding this interaction may provide new neurophysiological insights for optimizing visual interface design in SSVEP-based BCIs.

## 2. Materials and Methods

### 2.1. Participants

A total of 22 volunteer subjects (16 males and 6 females), aged between 19 and 30 years (mean age 24.7 ± 5.4 years), participated in this study. Recruitment was conducted at the Universidad de Miguel Hernandez de Elche (UMH) in Spain. As inclusion criteria, all participants were required to have normal or corrected-to-normal vision and report no history of epilepsy, photosensitivity, or other neurological disorders. In addition, the selected subjects reported having little to no prior experience using BCI systems. Participation was strictly voluntary, and the subjects were not financially compensated.

The design and execution of the experimental protocol were conducted in full compliance with the ethical principles established in the Declaration of Helsinki. Prior to the onset of any procedure, all participants were duly informed about the nature and objectives of the study and provided their written informed consent. To safeguard the privacy of the volunteers, all personal information was dissociated from the physiological recordings, ensuring confidentiality and total data anonymity during the storage and subsequent analysis phases.

The EEG recording sessions were conducted in a quiet and distraction-free laboratory environment. Although the room lacked electromagnetic shielding, environmental conditions were strictly controlled. In particular, room luminance was kept deliberately low and constant to optimize the contrast of the stimuli on the screen and minimize visual interference or fatigue for the participants.

### 2.2. Hardware and Data Acquisition

**Figure 1:**
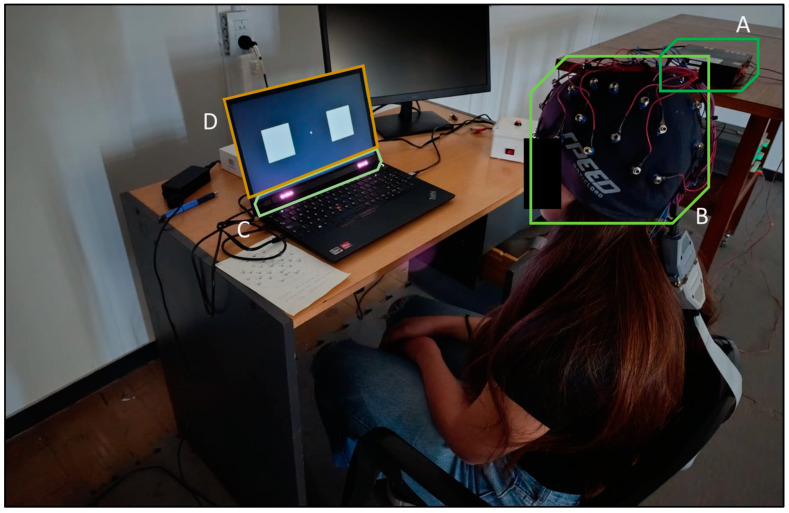
Experimental setup for the simultaneous acquisition of EEG signals and eye tracking: (A) RHD2132 electrophysiological signal acquisition and amplification system (Intan); (B) EEG recording cap (30 channels); (C) Joaco1 eyetracker (Neufitech); (D) presentation screen for the flickering visual stimuli (left square at 20 Hz, right square at 30 Hz).

The general layout of the experimental environment and the equipment used are illustrated in Figure 1. Participants sat at a distance of approximately 60 cm in front of a 15.6-inch LCD screen (Figure 1D) of a Lenovo ThinkPad E15 Gen 3 laptop (Lenovo, Bratislava, Slovakia). This screen was configured with a resolution of 1920 × 1080 pixels and a vertical refresh rate of 60 Hz. The experiment was executed on a 64-bit Windows 10 Home operating system (Microsoft Corporation, Redmond, WA, USA), powered by an AMD Ryzen 5 5500U processor at 2.10 GHz (Advanced Micro Devices, Santa Clara, CA, USA).

For EEG acquisition, an RHD2132 system (Intan Technologies, Los Angeles, CA, USA) was used, featuring 32-channel amplifiers and configured with a sampling frequency of 1000 Hz (Figure 1A). Thirty standard Ag/AgCl signal electrodes were mounted on a recording cap (Figure 1B) and distributed over the scalp according to the international 10–20 system. The ground electrode was attached to the earlobe, using the Cz position as a reference. Standard conductive electrolyte gel was applied between the electrodes and the scalp to maintain impedances below 10 kOhms at all times. The amplifier applied an analog band-pass filter from 1 to 50 Hz and a 50 Hz notch filter to attenuate power line interference.

Simultaneously, the gaze focus location was recorded using a Joaco1 model eyetracker device (Neufitech, Bahía Blanca, Argentina) (Figure 1C). This hardware, featuring compact dimensions (29.5 cm long by 1.4 cm high), was attached to the bottom edge of the screen pointing directly at the user’s face, and was connected to the computer via a USB 3.0 Type-C port. The device captures data at a rate of 60 frames per second (FPS) and is equipped with a camera and an infrared lighting system that guarantees stable tracking under various lighting conditions. Prior to the experimental tasks, the calibration interface provided by the manufacturer was executed to validate the spatial position of the device and identify the unique ocular characteristics of each subject, thereby maximizing tracking accuracy during visual stimulation.

### 2.3. Experimental Design

The experimental design consisted of a sustained visual attention task based on a simultaneous stimulation paradigm. Two visual stimuli (flickering white squares) were presented on a completely black background. Luminance modulation for both stimuli was performed using an on/off square wave. The square located in the left visual hemifield flickered at a constant frequency of 20 Hz, while the square located in the right hemifield flickered at 30 Hz. Spatially, the stimuli were aligned on the vertical equator of the screen (50% of the height). Horizontally, the center of the left square was positioned at 25% of the screen width, the central fixation cross at 50%, and the center of the right square at 75%. Considering the physical dimensions of the monitor and the viewing distance of 60 cm, this equates to an approximate eccentricity of 8.2 degrees of visual angle for each stimulus relative to the center. This spatial separation was strategically selected to exceed the 4.5-degree threshold, a distance below which concurrent visual stimuli have been shown to suffer competitive suppression in the early visual cortex [12]. Furthermore, the design employed large stimuli (subtending up to 6.9 degrees), as increasing the dimensions of the flickering field has been shown to be a key factor in maximizing SSVEP response amplitude [13].

To evaluate the impact of stimulus dimension on the cortical response, the test was divided into three consecutive blocks, evaluating three sizes: Small (200 × 200 pixels, subtending ~3.4 × 3.4 degrees of visual angle), Medium (300 × 300 pixels, ~5.1 × 5.1 degrees), and Big (400 × 400 pixels, ~6.9 × 6.9 degrees).

The temporal structure of the protocol was organized into “cycles”, each comprising three stimulation trials or “pulses”. Immediately before the onset of each pulse, a fixation cross was presented to explicitly indicate to the participant the exact spatial location where they should maintain their gaze during the subsequent stimulation block. To mitigate potential effects of neural habituation or temporal anticipation, the flicker duration was set pseudo-randomly within an interval of 4 to 8 s. Introducing this temporal variability prevents the nervous system from extracting precise statistical regularities [14]. In this way, proactive mechanisms of temporal expectation are prevented from building predictive models that attenuate sensory processing through habituation, thereby ensuring an optimal level of sustained attention [15]. This duration was kept constant for the three pulses within the same cycle to ensure comparability, but varied dynamically between cycles to maintain the user’s state of alertness. Between each stimulation pulse, a 5 s rest period with a black screen was introduced, allowing participants to relax their eyes and facilitating the return of electrophysiological activity to its baseline.

**Figure 2:**
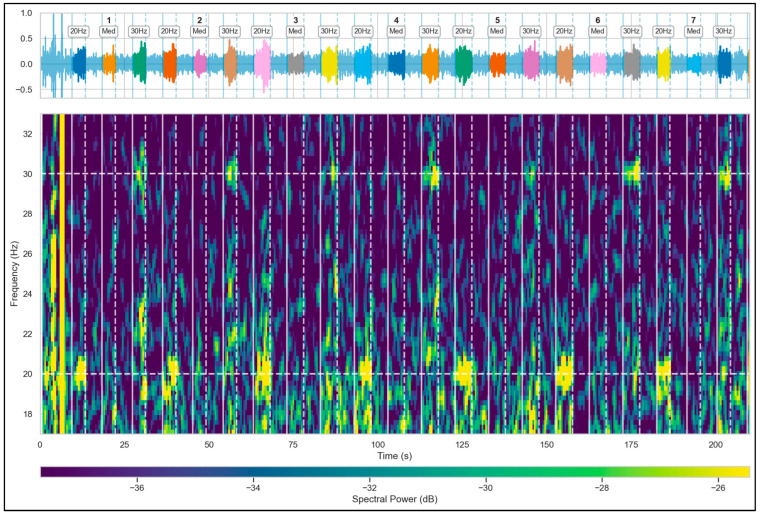
Temporal dynamics of the evoked cortical response during the experimental paradigm (Subject 20, POz channel, Medium size). A segment of the EEG signal and its synchronized spectrogram are illustrated, highlighting the amplitude fluctuations and the evolution of frequency energy density during the alternation between stimulation windows and rest periods.

During each cycle, participants were instructed to modulate their overt attention across three sequential states, guided by the preceding fixation cross. In the first pulse, the participant fixed their gaze on the center of the left square (the “20 Hz” state). Following the rest, in the second pulse, fixation was directed to the exact center of the black screen (the state of relative visual rest or “Med”), perceiving both flickering stimuli exclusively peripherally. Finally, in the third pulse, gaze was maintained on the center of the right square (the “30 Hz” state). A visual representation of this temporal structure and the corresponding evoked spectro-temporal response is presented in Figure 2. Throughout the execution of the protocol, the eye tracker device continuously recorded ocular kinematics. These optometric data were used in an offline processing stage to identify and discard from the final analysis any trials where the subject deviated their gaze from the indicated target or presented fixation issues that compromised the study design.

Each participant completed a total of 11 cycles per size block, yielding a total of 33 cycles (99 pulses) per recording session. The presentation order of the size blocks was kept fixed for all subjects following the sequence Small -> Medium -> Big. To prevent visual and cognitive fatigue, a mandatory 5 min rest period was established during the transition between each block.

### 2.4. Signal Processing and Feature Extraction

All offline data processing and spectral analysis were implemented using custom routines in Python (version 3.12.3), within the Jupyter Notebook development environment (version 7.0.8). The processing was carried out systematically, iteratively analyzing the data of each of the 22 subjects for the three stimulus size conditions (Small, Medium, Big).

#### 2.4.1. Eye-Tracking Validation and Spatiotemporal Preprocessing

Prior to the analysis of the EEG signals, the kinematic data from the eye tracker were synchronized and evaluated. This step was crucial to corroborate the strict adherence of each subject to the gaze focus instructions during the protocol; initially, 27 subjects were recorded, but 5 entire subject recordings were completely discarded due to significant gaze deviations, persistent uncorrectable EEG artifacts, or non-compliance with the task. This subject-level exclusion resulted in a final, perfectly balanced dataset of 22 subjects.

For the EEG signals, the first conditioning step consisted of spatial re-referencing using the Common Average Reference (CAR) technique. This method calculates the average of the instantaneous activity of the 30 channels and subtracts this value from each individual channel. This spatial filtering technique attenuates common-mode noise and enhances topographical resolution, improving SSVEP signal isolation over occipital and parieto-occipital regions [16,17]. Next, an optimal-order Butterworth digital band-pass filter between 15 and 40 Hz was applied to isolate the frequency band of interest and eliminate low-frequency drifts and high-frequency muscle noise. Subsequently, the continuous signals were segmented according to the event markers into windows corresponding to each stimulation pulse (20 Hz, Med, 30 Hz) across the 11 cycles of each recording. Finally, to enable a robust inter-subject comparison and nullify absolute impedance biases, the segmented data were normalized using the standard *Z*-score transformation.

#### 2.4.2. Spectral Parameterization

The frequency domain analysis was centered on the channels of primary visual interest (POz, O1, and O2). To estimate the Power Spectral Density (PSD), Welch’s method was applied using overlapping windows (75% overlap) to ensure optimal spectral resolution and reduce estimation variance within the flat passband region, carefully avoiding the filter’s roll-off edges.

To achieve a precise dissection of neural activity, the SpecParam (formerly known as FOOOF) spectral parameterization algorithm was implemented. Complete details regarding the algorithm settings, justification of the selected parameters, and model-fit quality metrics are provided in the Supplementary Material. This tool allowed modeling the empirical PSD and decomposing it into two physiologically distinct elements:

• The aperiodic component (1/f noise floor): The aperiodic decay model was calculated by fixing the fitting mode (linear fit in the log-log domain), from which the spectral exponent parameter (aperiodic slope) was directly extracted. This fixed approach is the optimal method for narrow, high-frequency bands where the aperiodic ‘knee’ is absent.
• The periodic component (SSVEP peaks): The genuine power of the evoked response was quantified by identifying the maximum energy peak within a narrow bandwidth of ±0.5 Hz around the target frequencies (20 Hz and 30 Hz). This specific window size was strictly dictated by the spectral resolution of our Welch’s estimation (0.5 Hz per frequency bin), limiting the search exclusively to the central target bin and its immediate adjacent neighbors. Furthermore, because SSVEPs naturally manifest as highly sharp, narrow-band responses, this constrained window prevents the erroneous integration of adjacent unspecific spectral noise that a broader threshold (e.g., ±1 Hz) might capture.

**Figure 3:**
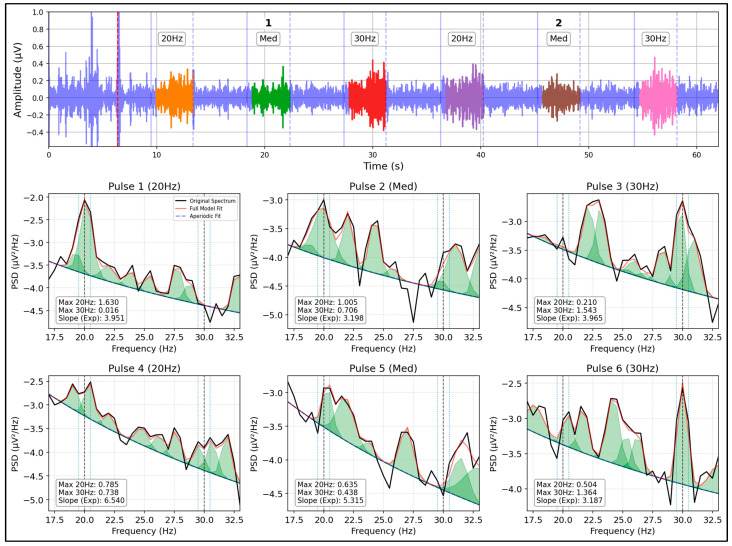
Spectral parameterization of the EEG signal using SpecParam (Subject 20, POz channel, Medium size). The decomposition of the PSD for the first six pulses of the experiment is illustrated, separating the total spectrum into its periodic component (SSVEP peaks at 20 and 30 Hz) and its aperiodic component (1/f background noise) to isolate pure oscillatory energy.

A fundamental aspect of this methodology was subtracting the modeled aperiodic noise floor from the total spectrum. Therefore, the SSVEP amplitude reported in this work strictly represents the pure oscillatory energy rising above the background neural activity, thus eliminating the confounding factors typical of traditional power analysis. The practical result of this spectral decomposition and modeling process, applied independently to each stimulation window, is illustrated in Figure 3.

Finally, the extracted metrics (maximum SSVEP powers and aperiodic slopes) from all pulses, cycles, channels, and conditions were consolidated into a global data matrix (dataframe), forming the definitive dataset for modeling and inferential statistical analysis at the population level.

### 2.5. Statistical Analysis

In order to ensure analytical rigor and avoid pseudoreplication, the metrics extracted from the 11 stimulation cycles were averaged intra-subject. Thus, a single representative value was obtained per participant (N = 22) for each combination of stimulus size (Small, Medium, Big), attention state (20 Hz, Med, 30 Hz), and recording channel (POz, O1, O2). This aggregation procedure was applied to both the power of the periodic signal (SSVEP) and the slope of the aperiodic component.

Given the repeated-measures experimental design (all subjects underwent all size conditions), inferential evaluation was conducted using non-parametric statistical tests. To analyze the main effect of the “Stimulus Size” factor on the dependent physiological variables, the Friedman omnibus test (a non-parametric alternative to the one-way repeated-measures ANOVA) was applied. This test was executed systematically and independently to evaluate the periodic response of the attended stimulus (Target), the response of the unattended stimulus (Distractor), and the dynamics of the aperiodic slope.

In scenarios where the Friedman test revealed a statistically significant main effect (rejection of the overall null hypothesis), a post hoc analysis was conducted to identify specific differences between the factor levels. For these pairwise comparisons (Small vs. Medium, Medium vs. Big, and Small vs. Big), the Wilcoxon signed-rank test was employed, and the resulting *p*-values were rigorously adjusted for multiple comparisons using the Bonferroni correction.

The significance threshold to determine the statistical validity of the findings was established a priori at a value of α = 0.05. All inferential statistical calculations were executed programmatically using the specialized scipy.stats module from the Python scientific ecosystem.

## 3. Results

### 3.1. SSVEP Power Dynamics and Spatial Selectivity (Periodic Component)

To evaluate the impact of stimulus size on the energy of the cortical response, the maximum spectral density of the periodic components (SSVEP peaks) was analyzed by extracting the aperiodic noise floor. The population analysis (N = 22) distinguished between the response evoked by the overtly focused stimulus (Target) and that generated by the unattended peripheral flickering stimulus (Distractor).

**Figure 4:**
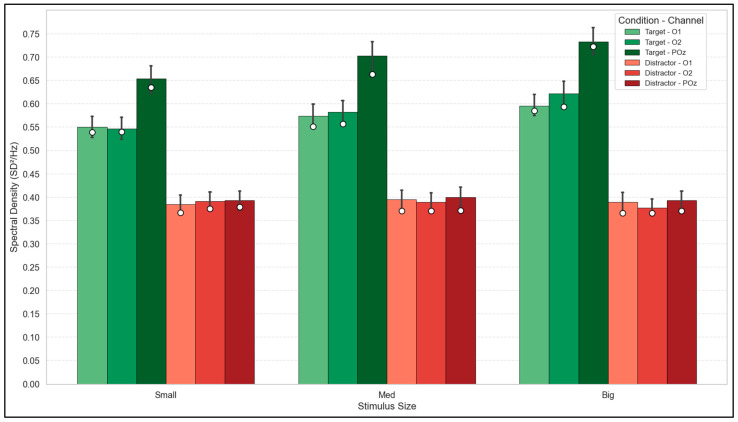
Population-level spectral density analysis of the periodic SSVEP response by stimulus size and channel. The extracted amplitudes for the attended stimulus (Target) are contrasted against the simultaneous peripheral stimulus (Distractor).

As illustrated in Figure 4, the increase in visual stimulus size induced a systematic increase in the power of the Target signal. Inferential evaluation using the Friedman test confirmed a highly significant main effect of the size factor on the amplitude of the attended signal across the three evaluated channels (*p* < 0.001). The Wilcoxon post hoc analysis corroborated that this power gain is significant when comparing the extreme conditions (Small vs. Big, *p* < 0.05), with this absolute increase being markedly higher in the occipito-parietal midline (POz channel). In contrast, the amplitude of the response evoked by the Distractor remained stable and at low levels across all spatial dimensions, showing no statistically significant modulation with increasing size (Friedman, *p* > 0.05). The observed convergence between the mean and the median for the Target condition confirms that this focalized power gain is a robust population phenomenon and not the artifact of outliers.

### 3.2. Modulation of the Aperiodic Component (1/f)

**Figure 5:**
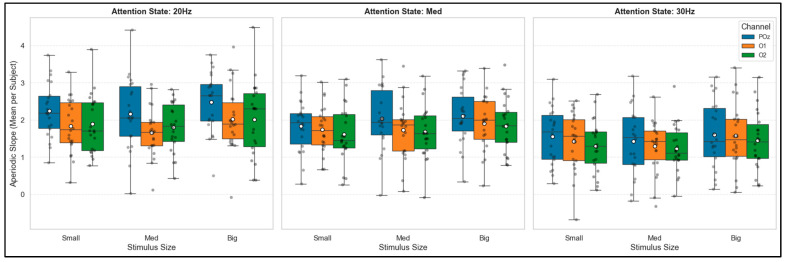
Population distribution of the aperiodic slope (1/f) as a function of stimulus size, stratified by attention state.

Beyond the fundamental oscillations, the dynamics of the background neural noise were evaluated through the analysis of the aperiodic slope (1/f spectral exponent). The results revealed that stimulus size acts as a significant modulator of this intrinsic activity, although its effect proved to be strictly dependent on the underlying stimulation frequency and attention state (Figure 5).

During the low-frequency attention state (20 Hz) and in the relative visual rest state (Med), increasing the stimulus size caused a statistically significant increase in the overall aperiodic slope (Friedman, *p* < 0.05). Specifically on the channel with the highest response (POz), the Wilcoxon post hoc analysis confirmed significant differences when scaling the stimulus to its maximum dimension (Small vs. Big, *p* < 0.05). This increase in the spectral exponent translates into a steeper drop in the curve, reflecting a reduction in high-frequency noise power. In contrast, during the 30 Hz attention state, the architecture of the aperiodic component remained statistically invariant to the geometric changes in the stimulus (Friedman, *p* > 0.05), maintaining a stable exponent across the three spatial conditions.

## 4. Discussion

The purpose of this study was to determine how the manipulation of the spatial dimensions of flickering stimuli modulates the full spectral dynamics of the EEG in a competitive stimulus SSVEP-BCI paradigm. The findings reveal that stimulus size operates through a synergistic dual mechanism: it not only enhances the amplitude of the fundamental oscillation but, under certain conditions, also restructures the background aperiodic activity.

First, the response of the periodic component demonstrates the extraordinary spatial filtering capacity of the human visual system. The fact that the geometric increase in the stimulus exclusively increases the power of the Target without inducing a simultaneous increase in the Distractor refutes the technical concern that stimuli of high eccentricity would “contaminate” the signal by invading peripheral receptive fields. The parieto-occipital cortex (POz) appears to spatially integrate information from the attended stimulus while maintaining robust tonic suppression over unattended elements.

The most novel finding lies in the reconfiguration of the aperiodic component. In recent neurophysiological literature, an increase in the slope of the 1/f spectrum has been validated as a biomarker of a shift in the E/I balance toward a state of greater GABAergic inhibition. In our paradigm, the increase in the aperiodic slope during 20 Hz attention and in the Med state when using larger stimuli suggests that the brain recruits active mechanisms of broadband noise suppression (lateral or functional inhibition) to facilitate neuronal synchronization in the face of an intense visual load. This active suppression of background noise enhances the overall SNR.

However, it is imperative to acknowledge that the 1/f exponent is a multifaceted metric. While our within-subject design inherently controls for macroscopic variables (such as age and baseline neuroanatomy), dynamic state-dependent alternative interpretations remain plausible. Beyond local GABAergic inhibition, a steeper high-frequency aperiodic slope can also reflect a widespread decrease in the asynchronous population spiking rate across the underlying cortical network. Additionally, the intense visual load imposed by larger stimuli may demand variations in general visual attention allocation or cognitive effort, which can independently modulate background aperiodic dynamics. Thus, while the homeostatic inhibition hypothesis provides a parsimonious explanation, these complementary mechanisms likely operate synergistically during visual processing.

The absence of this inhibitory modulation at 30 Hz is intriguing. One possible interpretation is that the neural networks responsible for processing higher frequencies operate in a resonance regime close to the early gamma band, where the fractal architecture of the neural noise reaches a “ceiling” of homeostatic inhibition, becoming insensitive to additional increases in spatial luminous energy.

It is important to note from a psychophysical perspective that in standard monitor-based stimulation, an increase in geometric stimulus size inherently entails a proportional increase in total luminous and contrast energy. Therefore, while the term “size” acts as a practical engineering parameter in BCI design, the observed neurophysiological phenomena are fundamentally driven by these physical sensory constraints. Specifically, the enhanced periodic signal is the direct result of a larger retinal stimulation area recruiting more extensive retinotopic networks, whereas the steepening of the aperiodic slope likely reflects a homeostatic inhibitory response triggered by the greater influx of total luminous energy.

While our current approach successfully isolates the periodic and aperiodic components using frequency-domain parameterization, it is important to acknowledge alternative and competitive modeling methods. Traditional spectral analysis assumes signal stationarity within the analyzed windows, which may not fully capture the rapid temporal fluctuations of brain wave dynamics. More recently, advanced mathematical frameworks, such as the multichannel dynamic modeling of non-Gaussian mixtures, have been proposed to characterize the continuous activity and functional connectivity of brain regions [18]. These models have proven highly effective in stimulus-response paradigms, including memory and learning tasks involving visual and auditory stimuli. In the context of SSVEP-BCIs, applying such dynamic modeling could theoretically allow for tracking the real-time non-stationary transitions of the E/I balance during the onset and offset of visual stimulation. This would offer a deeper understanding of the cortical network dynamics and connectivity changes induced by stimulus size, providing a complementary perspective to the static spectral analysis employed in this study.

Beyond these analytical considerations, a primary methodological limitation regarding our experimental protocol is the fixed presentation order of stimulus sizes (Small to Big). Although rest periods were included to reduce visual fatigue, future studies should use a counterbalanced design to eliminate possible order effects. Furthermore, the exploration of a broader spectrum of frequencies and their harmonics will be vital to determine exactly at which frequency limit this size-induced inhibitory modulation capacity is lost. However, this future line of research faces several practical constraints. First, there is an inherent technical limitation: the variety and precision of frequencies that can be evaluated are strictly constrained by the refresh rate of the monitor employed (e.g., 60 Hz), which restricts the stimulation to exact integer divisors to avoid temporal artifacts. Second, incorporating a wider frequency sweep inherently prolongs the experimental sessions, increasing the risk of visual fatigue. Finally, while lower frequencies generally elicit stronger responses, they also induce greater visual discomfort, particularly when combined with large stimulation areas, highlighting the ongoing trade-off between neurophysiological exploration and BCI user ergonomics.

Regarding the analytical pipeline, because our spectral parameterization was specifically restricted to the 15–40 Hz range to avoid low-frequency noise and the aperiodic ‘knee’, the estimated 1/f exponent strictly reflects high-frequency aperiodic dynamics. While this high-frequency slope is a validated biomarker of local functional inhibition, future studies utilizing broader spectral ranges (unconstrained by narrow band-pass filtering) are needed to determine if this size-induced modulation extends to the global broadband aperiodic component. Furthermore, the spatial analysis in this study was restricted to an a priori parieto-occipital Region of Interest (POz, O1, and O2). While this minimal channel configuration strictly aligns with the design principles of practical, user-friendly BCIs, it inherently precludes the assessment of widespread spatial dynamics. Future fundamental studies employing high-density EEG arrays and topographical mapping will be essential to visualize the full cortical distribution of this size-induced periodic enhancement and aperiodic inhibitory modulation. Additionally, since eccentricity remained constant in our study, future research should explore its interaction with stimulus size to determine how these neurophysiological effects generalize across different regions of the visual field.

Finally, while this study successfully isolated the fundamental sensory mechanisms driven by stimulus size, it did not incorporate subjective evaluations of visual fatigue or apply BCI performance metrics. Future translational research must evaluate how this size-induced enhancement in SNR translates into actual classification accuracy and ITR, while concurrently correlating these gains with subjective user experience questionnaires to fully validate their practical impact on BCI usability.

## 5. Conclusions

This work empirically confirms that the maximization of the SNR in SSVEP-BCI systems by increasing visual stimulus size is underpinned by a dual neurophysiological phenomenon. Traditionally, the improvement in the performance of these systems was attributed exclusively to the evocation of a more robust periodic signal, resulting from greater retinotopic recruitment. However, our results demonstrate that this enhancement of the fundamental signal is synergistically complemented by the interaction of a quieter neural background.

On the one hand, the analysis of the periodic component confirmed a high spatial selectivity of the visual system; increasing the size of the stimuli significantly increased the amplitude of the response to the attended target without exacerbating the interference of the peripheral distractor. On the other hand, the most revealing finding demonstrates that larger stimuli actively modulate intrinsic activity, inducing a steeper aperiodic (1/f) slope during 20 Hz attention and in visual rest periods. This attenuation of broadband noise reflects an effective recruitment of cortical inhibition mechanisms that stabilize the spectral environment. Notably, this inhibitory effect proved to be frequency-dependent, being absent at 30 Hz, which raises new and intriguing questions about the resonance limits of neural networks.

In summary, while the oscillatory component raises the “ceiling” of the useful signal, the aperiodic inhibitory modulation reduces the “floor” of the underlying noise. Taken together, these findings position stimulus size not merely as a geometric usability variable, but as a critical parameter for the engineering of robust interfaces.

## Data Availability

The data presented in this study are available on request from the corresponding author due to the data is currently being used for ongoing research.

## Supplementary Materials

The following supporting information can be downloaded at: https://www.mdpi.com/article/10.3390/brainsci16040424/s1, File: Spectral Parameterization (SpecParam) Settings and Quality Metrics.
