# Supplementary material for: Stimulus Size Modulates Periodic and Aperiodic EEG Components in SSVEP-Based BCIs
Source: Brain Sci. 2026 Apr 18;16(4):424. doi: 10.3390/brainsci16040424 (PMC13114236; doi:10.3390/brainsci16040424)
Supplement: Supplementary file 1 [file brainsci-16-00424-s001.zip › brainsci-4227740-supplementary.pdf]

Supplementary Material

Article:

Stimulus Size Modulates Periodic and Aperiodic EEG Components in SSVEP-Based BCIs

Gerardo Luis Padilla and Fernando Daniel Farfán

Spectral Parameterization (SpecParam) Settings and Quality Metrics:

To isolate pure oscillatory activity from background neural noise, the Power Spectral Density (PSD) of each stimulation window was decomposed using the SpecParam algorithm. The model was applied across the 15 to 40 Hz frequency range, corresponding to the digital band-pass filter previously applied to the Electroencephalography (EEG) signals. The specific hyperparameters used for the algorithm, along with their neurophysiological and computational rationale, were established as follows:

- **Aperiodic Mode:** "fixed"
  Rationale: The analysis was restricted to a relatively narrow, high-frequency band (15 to 40 Hz). Within this specific range, the aperiodic component of the EEG spectrum does not typically exhibit a "knee" (which is usually found at lower frequencies). Therefore, modeling the background noise as a linear fit in the log-log space (the fixed mode) is the standard and mathematically optimal approach for this bandwidth.

- **Peak Width Limits:** [0.8, 3.0] Hz
  Rationale: Steady-State Visual Evoked Potentials (SSVEPs) elicited by highly controlled flickering stimuli present as extremely sharp, narrow-band frequency peaks. The lower bound of 0.8 Hz prevents the algorithm from overfitting spurious, infinitely narrow noise spikes. Conversely, the upper bound of 3.0 Hz ensures that the model specifically targets these sharp SSVEP components, preventing the algorithm from erroneously fitting broader, unspecific physiological oscillations.

- **Minimum Peak Height:** 0.01
  Rationale: This threshold was set conservatively low to ensure that subtle evoked responses, particularly those generated by the unattended peripheral stimuli (Distractor condition), could be detected and quantified reliably without forcing the model to fit the absolute baseline noise.

- **Peak Threshold:** 0.05 (standard deviations)
  Rationale: This parameter defines the threshold for detecting peaks above the aperiodic background. A relatively permissive value of 0.05 was selected to maximize the sensitivity to small but genuine periodic responses. The risk of false positives was effectively mitigated by the strict "Peak Width Limits" constraint described above.

Model-Fit Quality Metrics: To guarantee the reliability of the spectral decomposition, goodness-of-fit metrics were extracted for all computed models. The algorithm automatically calculates the R-squared value and the Mean Absolute Error for each fit, comparing the modeled spectrum against the empirical PSD. The convergence of the model and the robustness of the extracted

parameters (periodic amplitude and aperiodic slope) were confirmed to be consistent across all subjects and experimental conditions.
